# Supplementary material for: Cu-Doped Boron Nitride Nanosheets for Solid-Phase Extraction and Determination of Rhodamine B in Foods Matrix
Source: Nanomaterials (Basel). 2022 Jan 19;12(3):318. doi: 10.3390/nano12030318 (PMC8838717; doi:10.3390/nano12030318)
Supplement: Supplementary file 1 [file nanomaterials-12-00318-s001.zip › nanomaterials-1506855-supplementary.pdf]

<Supporting Information>

Cu doped boron nitride nanosheets for solid-phase extraction and determination of Rhodamine B in foods matrix

Fujie Liu [1], Qihang Zhou [2], Yurui Li [2], and Jingyu Pang [2,*]

[1] Zhenjiang Key Laboratory of Functional Chemistry & Institute of Medicine and Chemical Engineering, Zhenjiang College, Zhenjiang 212028, P. R. China, fjie520@163.com

[2] Henan Key Laboratory of Polyoxometalate Chemistry, College of Chemistry and Chemical Engineering, He-nan University, Kaifeng 475004, P. R. China, zhouqh0717@163.com (Q.Z.); L2848781123@163.com (Y.L.)

* Correspondence: pjy@henu.edu.cn. Tel.: +86-0371-23881589

Contents:

Section 1 Supplementary Tables (1-5).

Section 2 Supplementary Figures (1-2).

Table S1 kinetics parameters of RhB adsorbed onto Cu (1%)-BN.

	Pseudo-first order			Pseudo-second order			
	q_e (mg/g)	K_1 (min^{-1})	R^2	q_e (mg/g)	$t_{1/2}$ (min)	K_2 ($g\bullet mg^{-1}min^{-1}$)	R^2
BN	235	0.042	0.9591	417	2.92	0.00082	0.9988
Cu(0.1%)-BN	193	0.048	0.7823	526	1.63	0.0012	0.9999
Cu(0.2%)-BN	179	0.036	0.6766	556	1.33	0.0013	0.9996
Cu(0.5%)-BN	274	0.039	0.9414	500	2.70	0.00074	0.9986
Cu(1%)-BN	258	0.044	0.9553	476	2.57	0.00082	0.9989
Cu(2%)-BN	120	0.038	0.7343	333	1.60	0.0019	0.9997

Table S2 Isotherm parameters of RhB adsorption onto Cu (0.2%)-BN at 298 K.

	Langmuir				Freundlich			
	q_m (mg/g)	K_L(L/mg)	R^2	APE%	K_F (L/mg)	n	R^2	APE%
RhB	743	0.22	0.9941	1.21	5.92	334	0.8932	3.36

Table S3 Comparison of the BN adsorption capacity of RhB with other literatures.

**Adsorbents**	**q_m**	Ref.
cotton flower-like hierarchically porous boron nitride	313.4	[1]
hexagonal boron nitride nanosheets	16	[2]
Porous hexagonal boron nitride whiskers	210.1	[3]
boron nitride nanosheets	75.65	[4]
Few-Layer Boron Nitride	488	[5]
Flake Boron Nitride	125	[6]
boron nitride nanosheets	124	[7]
Cu doped boron nitride nanosheet	743	This work

q_m: the theoretical maximum adsorption capacity calculated by adsorption isotherms model.

Table S4 Thermodynamic parameters for the RhB adsorption onto Cu(0.2%)-BN.

Adsorbent	ΔG (KJ/mol)				ΔH (KJ/mol)	ΔS (J/(mol•K))
	289 K	308 K	318 K	328 K		
BN	-1.19	-0.76	-0.36	-0.0084	-12.93	-39
Cu(0.2%)-BN	-9.86	-8.85	-7.91	-6.8	-39.9	-101

Table S5 Comparison of the current method with other reported methods.

Sample	Method	Recovery (%)	LOD	LOQ	RSD (%)	Ref.
Hamoon lake water	DLLME	99.62 ± 1.00	50 ng/mL	145 ng/mL	0.9-3.1	[8]
Karoon river water	MSPE	95.7	5.91 ng/mL	-	1.1-4.1	[9]
hand washing liquid soap	CPE–spectrophotometry	96-103.5	1.3 μg/L	5-550 μg/L	0.5-1.6	[10]
Red wine	SPE-HPLC	70-75	250 μg/L	1-10 μg/mL	2.87	[11]
Dish washing liquid	M'SPE	95.74±4.59	0.1 μg/L	0.35-5 μg/L	2.1-8.2	[12]
Drinks	SPE-HPLC	89.8-95.4	0.028 ng/mL	0.092 ng/mL	3.0-4.3	This work

DLLME: Dispersive Liquid–Liquid Micro-extraction; MSPE: magnetic solid phase extraction; M'SPE: micro solid phase extraction; SPE: solid phase extraction; CPE: cloud point extraction.

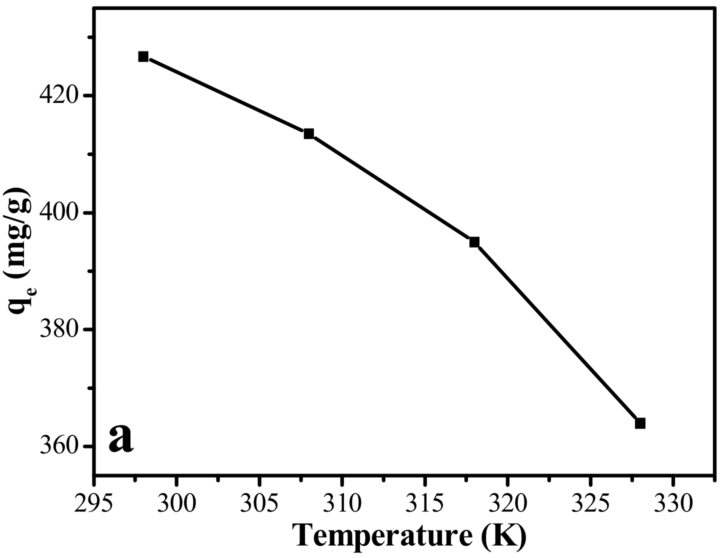

Figure S1. Effect of temperature on the adsorption of RhB onto the Cu(0.2%)-BN

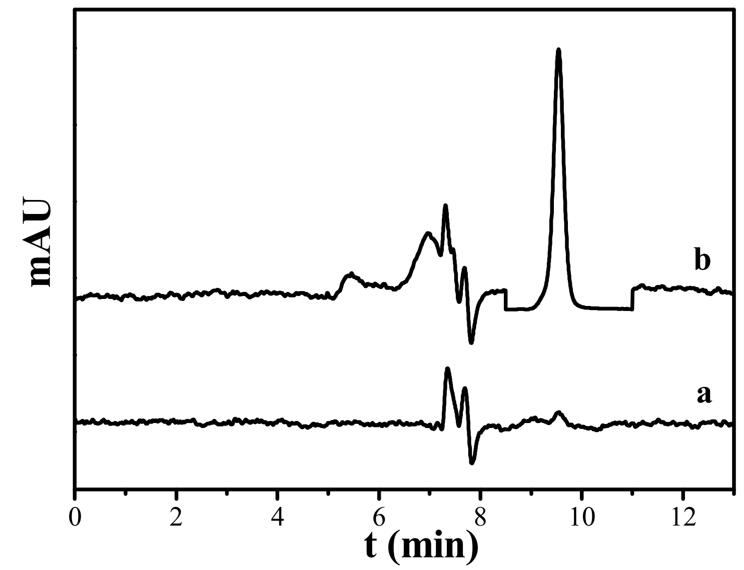

Figure S2. HPLC results. (a) sample 1, (b) spiked sample (500 ng/mL).

Reference

1. Maiti, K., Thanh, T. D., Sharma, K., Hui, D., Kim, N. H., and Lee, J. H., Highly efficient adsorbent based on novel cotton flower-like porous boron nitride for organic pollutant removal. *Composites Part B-Engineering* **2017**. *123*, 45-54.
2. Mahdizadeh, A., Farhadi, S., and Zabardasti, A., Microwave-assisted rapid synthesis of graphene-analogue hexagonal boron nitride (h-BN) nanosheets and their application for the ultrafast and selective adsorption of cationic dyes from aqueous solutions. *Rsc Advances* **2017**. *7*, 53984-53995.
3. Li, Q., Yang, T., Yang, Q. F., Wang, F., Chou, K. C., and Hou, X. M., Porous hexagonal boron nitride whiskers fabricated at low temperature for effective removal of organic pollutants from water. *Ceramics International* **2016**. *42*, 8754-8762.
4. Wang, X. B., Yang, Y. F., Jiang, G. D., Yuan, Z. W., and Yuan, S. D., A facile synthesis of boron nitride nanosheets and their potential application in dye adsorption. *Diamond and Related Materials* **2018**. *81*, 89-95.
5. Chang, H. H., Chao, Y. H., Pang, J. Y., Li, H. P., Lu, L. J., He, M. Q., Chen, G. Y., Zhu, W. S., and Li, H. M., Advanced Overlap Adsorption Model of Few-Layer Boron Nitride for Aromatic Organic Pollutants. *Industrial & Engineering Chemistry Research* **2018**. *57*, 4045-4051.
6. Qu, J. L., Li, Q., Lu, C., Cheng, J., and Hou, X. M., Characterization of Flake Boron Nitride Prepared from the Low Temperature Combustion Synthesized Precursor and Its Application for Dye Adsorption. *Coatings* **2018**. *8*.
7. Chao, Y., Liu, M., Pang, J., Wu, P., Jin, Y., Li, X., Luo, J., Xiong, J., Li, H., and Zhu, W., Gas-assisted exfoliation of boron nitride nanosheets enhancing adsorption performance. *Ceramics International* **2019**. *45*, 18838-18843.
8. kakhki, R. M., Nejati-Yazdinejad, M., and Kakeh, F., Extraction and determination of Rose Bengal in water samples by dispersive liquid–liquid microextraction coupled to UV–Vis spectrophotometry. *Arabian Journal of Chemistry* **2017**, *10*, S2518-S2522.
9. Parham, H., Zargar, B., Heidari, Z., and Hatamie, A., Magnetic Solid-Phase Extraction of Rose Bengal Using Iron Oxide Nanoparticles Modified with

Cetyltrimethylammonium Bromide. *Journal of the Iranian Chemical Society* **2011**, *8*, S9-S16.
10. Pourreza, N., Rastegarzadeh, S., and Larki, A., Micelle-mediated cloud point extraction and spectrophotometric determination of rhodamine B using Triton X-100. *Talanta* **2008**, *77*, 733-736.
11. Xiao-Yan, L. I., Mei, L. I., Chen, Q. F., Wei, S. Y., Luo, Y., and Tong, H. J., Determination of Rhodamine B in Red Wine by Solid Phase Extraction-High Performance Liquid Chromatography. *Food Science* **2011**, *32*, 238-243.
12. Bagheri, H., Daliri, R., and Roostaie, A., A novel magnetic poly(aniline-naphthylamine)-based nanocomposite for micro solid phase extraction of rhodamine B. *Analytica Chimica Acta* **2013**, *794*, 38-46.
